# Chinese immigrant men smokers’ sources of cigarettes in Canada: A qualitative study

**DOI:** 10.1186/s12971-017-0123-1

**Published:** 2017-03-21

**Authors:** Aimei Mao, Joan L. Bottorff, John L. Oliffe, Gayl Sarbit, Mary T. Kelly

**Affiliations:** 1 Kiang Wu Nursing College of Macau, Est. Repouso No.35, R/C, Macau, China; 2 Institute for Healthy Living and Chronic Disease Prevention, University of British Columbia, Kelowna, Canada; 3 Faculty of Health Sciences, Australian Catholic University, Melbourne, Australia; 4 School of Nursing, University of British Columbia, Vancouver, Canada

**Keywords:** Chinese immigrants, Canada, Qualitative study, Sources of cigarettes

## Abstract

**Background:**

Immigrants often experience economic hardship in their host country and tend to belong to economically disadvantaged groups. Individuals of lower socioeconomic status tend to be more sensitive to cigarette price changes. This study explores the cigarette purchasing patterns among Chinese Canadian male immigrants.

**Methods:**

Semi-structured in-depth interviews were conducted with 22 Chinese Canadian immigrants who were smoking or had quit smoking in the last five years.

**Results:**

Because of financial pressures experienced by participants, the high price of Canadian cigarettes posed a significant challenge to their continued smoking. While some immigrants bought fully-taxed cigarettes from licensed retailers, more often they sought low-cost cigarettes from a variety of sources. The two most important sources were cigarettes imported during travels to China and online purchases of Chinese cigarettes. The cigarettes obtained through online transactions were imported by smoking or non-smoking Chinese immigrants and visitors, suggesting the Chinese community were involved or complicit in sustaining this form of purchasing behavior. Other less common sources included Canada-USA cross border purchasing, roll your-own pouch tobacco, and buying cigarettes available on First Nations reserves.

**Conclusions:**

Chinese Canadian immigrant men used various means to obtain cheap cigarettes. Future research studies could explore more detailed features of access to expose gaps in policy and improve tobacco regulatory frameworks.

## Background

Tobacco use is the most important preventable cause of morbidity around the world, and is associated with nearly six million deaths per year [1]. Smoking is disproportionately represented among people from lower social economic status (SES) [2–4]. People in lower income groups and with less education are more likely to buy cheaper cigarette brands [5–7]. As a result, these groups are more sensitive to increased taxes on tobacco products and more often attempt to access cheap cigarettes through unconventional channels, such as purchasing cigarettes in duty-free shops, buying trafficked untaxed cigarettes, and hand rolling tobacco leaves [8–10]. It is believed that the illicit tobacco market may account for as much as one in every 10 cigarettes consumed globally [1].

China has the largest number of smokers in the world - one in three smokers in the world is Chinese [11]. Smoking in China is predominantly a male behavior. More than half of Chinese adult men smoke while less than 3% of Chinese women smoke [11]. China is also the largest producer of cigarettes. Tobacco use in China is influenced by wide variation in cigarette prices, ranging from less than 10 RMB (Renminbi, Chinese dollars) to more than 1000 RMB a pack (20 cigarettes/pack and 10 packs/carton), thereby accommodating smokers from all income levels. Although the wholesale tax on cigarettes in China has increased from 5 to 11%, it still lags far behind the WHO (World Health Organization) recommended level of 67–80% [8] and makes cigarettes relatively cheap for most smokers.

Studies indicate that immigrants to industrialized countries often experience difficulty in finding suitable employment and usually obtain lower salaries than other citizens [12–14]. This difficulty is usually more apparent among new immigrants than long-term immigrants, but nonetheless immigrants often belong to social and economically disadvantaged groups [7, 15–17]. Inversely, new immigrants from cultures with high smoking prevalence also smoke more than older immigrants and mainstream populations before they adapt their smoking patterns to the norms of the new environment [18].

Chinese male smokers may experience challenges to their continued smoking after they immigrate to developed countries, given the economic difficulties they encounter, combined with the probability of higher priced cigarettes in the host country. Studies on Chinese immigrants’ smoking behaviors are limited. Several studies, mainly from the United States, report that Chinese immigrant smokers reduce their smoking more than other subpopulations [19–22]. Despite reductions, a substantial proportion of Chinese immigrants continue to smoke [23]. Poorly understood are the influences of different tobacco markets in the host countries on Chinese immigrants’ smoking.

This article is drawn from a qualitative study focused on the smoking behaviors of Chinese Canadian immigrant fathers who smoke [24, 25]. As part of the study, participant’s access to cigarettes was investigated and these data were analyzed for the current article. Canada has a history of introducing comprehensive tobacco control measures and has continued to increase taxation on tobacco products. As a result, Canada has one of the lowest smoking rates in the world, with a general smoking prevalence of 14.6% (16.0% among men and 13.3% among women) [26].

From 2006 to 2015, 290,912 new permanent residents from China landed in Canada, and the Chinese comprise the second largest foreign-born group in Canada [27]. It is important to explore how cigarettes are accessed to inform tobacco control policy and the development of interventions to reduce smoking among Chinese immigrants.

## Methods

### Participant recruitment

A qualitative descriptive approach as described by Sandowski [28, 29] was used focusing on gaining a full understanding of behaviors and practices from participant perspectives. Bilingual recruitment advertisements were distributed to Chinese organizations in the lower mainland of British Columbia, Canada and posted on Chinese online forums. The forums were publically accessible and hosted on websites in Canada with Chinese language to serve Chinese Canadian immigrants. Potential participants were invited to contact the research team by telephone or email and then screened for eligibility.

Criteria for eligibility included the following: 1) self-identified as a male Chinese immigrant or Chinese Canadian; 2) currently smoked or quit smoking in the past five years; and 3) lived in Canada for at least half a year. As per the original project, participants were fathers who were expecting a child or had a child under the age of five years. Twenty-two men, recruited through the Chinese forums (*n* = 21) or through the Chinese organizations (*n* = 1), met the study criteria and provided informed consent. All participants were first-generation immigrants; two migrated to Canada with their parents before 18 years of age, and the other 20 migrated after age 18. They had lived in Canada for an average of 8.7 ± 6.4 years (range = 0.5 to 22 years), 19 of the men for less than 15 years. Characteristics of the sample are shown in Table 1.

**Table 1:** Demographics and smoking patterns of the Chinese immigrants who smoked

Category	Number of participants
Education	
Elementary school and below	0
Junior/middle school	1
High school	0
Non-university (collage, vocational, technical, trade etc.)	3
Bachelor’s degree	13
Master’s degree or over	5
Occupation	
Clerical/Administrative	5
Construction/Manual Labor	7
Technical/Skilled/Professional/Trade	7
Unemployed (Disabled, Student)	3
Marital status	
Married	21
Divorced	1
Amount Smoked	
≤ 10/day	7
10-20/day	3
> 20/day	0
Quit Smoking	12
Age (years old)	38 ± 5.0 (28 to 46)
Years in Canada	8.7 ± 6.4 (0.5 to 22)

All 22 participants were smoking at the time they migrated to Canada. At the time of interview, 12 participants had quit smoking (defined as having stopped smoking for at least one week) and the other ten had substantially reduced their smoking. Two of the ex-smokers quit their smoking around five years ago while the other ten quit within the last three years. Among the current smokers, three smoked 10–18 cigarettes per day (CPD) and the other seven smoked fewer than 10 CPD.

### Data collection

Semi-structured interviews were conducted via telephone with all the participants except one, with whom a face-to-face interview was conducted. Canada is a vast country in terms of geographical size and telephone interviewing maximized the researchers’ ability to reach Chinese immigrants from multiple regions. More importantly, telephone interviewing afforded participants more anonymity than face to face interviewing [30]. The interviews focused on changes in the participants’ smoking since immigration, exploring participants’ access to cigarettes. Interview questions included: “How has your life changed since you came to Canada?”; “How has your smoking changed since you came to Canada?” “What do you think of the differences in smoking practices between Canada and China?” and “How do you get your cigarettes in Canada?”

Probes and follow-up questions were used to encourage the participants to provide more information related to the purposes of the study. The interviews were conducted by the first author, a bilingual researcher (AM), and lasted from between 30 and 90 min. The data from the 22 interviews were rich, covering different aspects of the participants’ smoking experiences. The changed smoking behaviors and their related contributors, and the participants’ perception on the smoking difference between China and Canada have been presented in somewhere else [24, 25, 31]. Probes and follow-up questions were also used to encourage the participants to share information on whether and how they accessed cigarettes via unconventional ways, such as online purchases, duty-free purchases, and cross-border shopping, etc. The participants clearly related their accessing cigarettes to their economic situation and supplemented information concerning their employments to the question “How has your life changed since you came to Canada?” These data set foundation for this article.

### Data analysis

The participants, who were all Chinese immigrants currently smoking or having once smoked, were coded as “CS” (Chinese smoker), and numbered sequentially as they entered the study. All interviews were digitally recorded, translated into English and transcribed. A bilingual research assistant with Chinese and English proficiency translated the interviews and the translations were checked by the bilingual researcher (AM). The team used qualitative content analysis [31] wherein the primary aim was to describe the phenomena. The categories and names were initially derived from data based on close readings of the first three interviews. These emergent categories were organized and grouped into meaningful clusters and definitions developed to form a coding framework that was used to systematically code the interview data. The qualitative data management program NVivo8 was used to code and retrieve data. Data coded to each of the categories were reviewed in detail by comparing and contrasting data from all participants. Attention was paid to identifying patterns in participants’ perspectives about accessing cigarettes and illustrative quotes were identified.

## Results

The men quit or reduced their smoking due to various reasons, as ‘Concern over impacts of smoking on children’s health’, ‘Different smoking environment between China and Canada’, and ‘economic concern’ being cited as the most important facilitators. These facilitators have been presented elsewhere [24]. Although ‘economic concern’ was cited as an independent facilitator, this factor was, in fact, interplayed with other factors to produce complicated influence on the men’s changed smoking, which will be described in detail below.

Access to cigarettes and smoking patterns following immigration were directly influenced by challenges associated with immigration, the landscape of tobacco control in Canada and the responsibilities associated with fatherhood. The majority (20) of the 22 participants migrated to Canada in adulthood, and they described significant life changes after immigration. In most of the participants’ families, the wives were either full-time caregivers or worked part-time, because they could not afford paid childcare services. The fathers (participants) were expected to act as the primary breadwinners for the family. Despite being well educated and/or having had good jobs in China, the men had difficulty finding suitable jobs after they arrived in Canada. Several participants attended Canadian universities to pursue further career training. Others were underemployed, and worked as laborers or in unskilled jobs with poor salaries. Several participants worked two part-time jobs, mainly in Chinese restaurants or supermarkets.

All participants, both current smokers and ex-smokers, talked about the high price of cigarettes in Canada and in relation to their financial situation. They complained that the cigarettes in Canada were much more expensive than those in China. Unlike China, where a wide price range in cigarettes facilitates smoking for individuals from different economic conditions, Canadian cigarettes were universally expensive with no discounted or cheaper brands. Several participants mentioned going to licensed retailers, such as gas stations, supermarkets, or convenient stores, to buy full-taxed cigarettes, but more often they sought cheaper sources to afford their smoking (Table 2). The following section details the avenues participants found to purchase cheap cigarettes.

**Table 2:** The price-reduction sources cigarettes mentioned by the participants

The sources	N
Bringing in cigarettes from China	20
Online purchase	11
Buying cigarettes in duty-free shops	8
Cross US-Canada border purchase	7
Roll-your-own cigarettes	2
Purchase in Indian’ reservations	1

### Bringing in cigarettes from China

Almost all the participants described obtaining Chinese cigarettes directly from China. They did this for two reasons. The most prominent reason was, of course, the lower price of Chinese cigarettes. The other reason was that participants preferred the taste of Chinese cigarettes. A participant (CS2) who had lived in Canada for three years and had quit smoking for four months explained the taste difference between Chinese and Canadian cigarettes, *“Chinese cigarettes are toasted, while western cigarettes are mixed. The tastes are quite different.”* (CS2). The mixed cigarettes were those made of toasted and sun dried tobacco. Some participants, especially those who had lived in Canada for less than two years, complained about the “unpleasant” taste of Canadian cigarettes.

The participants, therefore, sought out Chinese cigarettes. Since Canadian customs allowed adults entering Canada to bring in one carton of cigarettes, participants commonly asked their friends and family members, whether they were smokers or not, to bring cigarettes whenever they visited Canada. One participant stated: *“When I first came to Canada… I brought a couple of cartons of Chinese cigarettes. Later my friends brought 5 or 6 for me when they came here from China.”*(CS2, ex-smoker). With the increasing number of Chinese individuals migrating to Canada and greater numbers of Chinese visitors entering Canada every year, global travel patterns between China and Canada have become an important means of supply for cheap cigarettes. A current smoker (CS14) who smoked 2–3 CPD and had lived in Canada for seven years said *“My source of Chinese cigarette never stops.”*

In China, smoking is embedded into men’s social life and smoking premier brands of cigarettes is a cultural signifier of status; however, once in Canada the participants no longer felt compelled to share and gift cigarettes with other men. A participant (CS12) who had quit smoking three years previous, described how Chinese immigrants usually did not buy the premier brands, but felt the ordinary Chinese cigarettes now met their needs, *“The expensive cigarettes in China, like Zhonghua* [the name of a premier brand of Chinese cigarettes] *is not fancy here because many Chinese don’t smoke that brand. The ordinary brands of 5 or 10 RMB* [1Canadian dollar =0.98U.S. dollar =6.04 Chinese renminbi in 2013] *a pack are popular.”* Participants who had once purchased premier brands to impress their business partners, superiors, and colleagues, thought they spent less on smoking after arriving in Canada. Also, because there was no culture of gifting tobacco in Canada, the participants could more easily estimate how long their cigarette stock would last in scheduling a fresh supply.

### Online purchase

The participants said they used the Internet to find online sources for Chinese cigarettes. With dozens of Chinese websites available in Canada, the participants never found difficulty in seeking out online sellers. Online sales were offered by both smokers and non-smokers and presented significant savings for regular smokers. A participant who had quit smoking one and half years ago said:

*Chinese cigarettes are much cheaper than Canadian cigarettes. I can tell you if Caucasians are able to buy cigarettes from China, they would buy them too. It is a competition. One pack of cigarette in Canada costs 8 or 9 dollars but it only costs 3 or 4 dollars for the same brand in China; it is almost half price. If you do the calculation, how much money can you save in a year if you save 30 dollars every month?* (CS8)

Because of Canadian customs laws, the online cigarette sales are for personal use only. The participants, however, had noticed how the online sales were expanding rapidly. *“The online market is indeed a big market, and a mature market. If you surf the XX* [one Chinese website in Canada, name redacted]*, you will find that many Chinese are selling cigarettes there.”* (CS6, current smoker)

The only concern participants expressed about online cigarette sales was the possibility of buying “fake” cigarettes. The Chinese immigrants were adamant that there were many fake cigarettes in the marketplace in China; therefore, it was possible that fake cigarettes would find their way to online markets. It is not surprising that the possibility of buying fake cigarettes prevented several participants from buying online. A participant (CS20) who currently smoked 1–3 CPD said, *“I don’t buy cigarettes shown on those Chinese websites, because my friends told me there are fake.”* Therefore, the online sales often involved face-to-face contact between buyers and sellers after initial contact had been established over the Internet. When personally meeting a seller, the buyer would have a chance to check whether the cigarettes were fake or not. A participant who currently smoked (CS 17) said he sometimes bought cigarettes online, and described his way of assessing the possibility of fake cigarettes: *“After we met he showed me the carton he was selling. I checked the packing and smelled the cigarettes. He encouraged me to try one of the cigarettes.”*

Despite being referred to as an “online” purchase by the participants, the Chinese websites actually functioned as a method of connecting buyers and sellers in person, because there was no payment system on the websites. Payment took place offline, face-to-face, as CS17, a current smoker, detailed about the purchasing process: “*You go to the web, find someone near your place selling cigarettes. You make a phone call. Then you decide to meet each other at some time in somewhere. You then do the trade face to face.”*

As importing cigarettes from China for personal use was legal, the online trade was also perceived by participants to be legal. A participant who had quit smoking for four months (CS2) but had previously accessed cigarettes using the online system, defended his purchase, saying: *“It is usually legal, unless you buy cigarette packs in a large number from someone.”* So it was possible that both sellers and buyers sometimes divided larger sales into smaller chunks to avoid being labeled as “illegal.”

### Buying cigarettes in duty-free shops

Duty-free shops were a must-go place to shop for almost all the participants when they travelled across borders. These shops are the only Canadian retail outlets that sell Chinese cigarettes, and the cigarettes in these shops are even cheaper than those in Chinese markets. A participant (CS12) who had quit smoking for three years spoke of the price difference, *“I bought a carton of Zhonghua* [a Chinese cigarette brand] *in a duty-free shop in the US. It was a little more than 40USD, cheaper than in China.”* Interestingly most participants, including those who had quit smoking, purchased duty-free cigarettes not for themselves but for other people, because the brands of cigarettes in the duty-free shops were often the premier ones. Also, the participants preferred the premier brands from duty-free shops because these brands were believed to be genuine; whereas, people might get fake brands in the markets in China. In fact, cigarettes in the duty-free shops were in such demand that it was often difficult for the participants to obtain them. One participant (CS12) remarked that although he had quit smoking, he still routinely went to the duty-free shops to buy cigarettes and other items for his family and friends each time he crossed the border. According to him, it was hard to get the premier cigarettes in the airports duty-free shops, but he always tried;

*You are not able to get them. They are sold out quickly. I guess it is because there are too many fake brands in Chinese markets. My friends who work in the airport told me that all the Zhonghua* [a premier cigarette brand] *would be bought up by the clerks working there as soon as Zhonghua arrived.* (CS12, ex-smoker)

Regardless of smoking status, most participants, as well as their friends and family members, always stopped in duty-free shops to purchase cigarettes for gifts or personal use. The cigarettes purchased in duty-free shops here went in two directions: inside Canada for immigrant smokers’ use and outside Canada to satisfy the Chinese gifting culture and international smokers’ preference for specific brands.

### US-Canada cross border purchases

Some participants described their purchase of cigarettes from US markets. They mentioned how cigarettes were cheaper in the US: *“My friends brought me Camel and Davidoff from the US. These are American brands. There are the same brands here in Canada. But they are cheaper in the US.”*(CS14, current smoker) *“One pack of Marlboro costs 11 dollars in Canada; if I go to the US I only spend 7 dollars for a pack.”* (CS13, ex-smoker)

Despite cheaper cigarettes, US-Canada cross border purchases were practiced by only a few participants due to customs laws. One participant explained, *“There is a regulation that you need to stay in the US for at least 48 h for being able to bring in a carton of cigarettes.”* (CS13, ex-smoker). As a result, the participants who smoked asked other people to buy cigarettes for them whenever they visited the US. Two participants who lived near the US border stated that US cigarettes were their primary source of cigarettes. They frequented the US for cheaper cigarettes and other daily necessities. One participant who had lived in Canada for 11 years and had quit smoking just two months previous due to health concerns for his two young children, joked that he crossed the border so often that he was more American than Canadian;

*I live in Surrey and it takes me only 20 min to drive to the Costco in the US. All the goods in the US supermarkets are cheaper than those in Canada. Once I run out of cigarettes I go there to buy. In daily life I spend USD not CAD [Canadian dollar]. I feel myself semi-American, perhaps more American than Canadian.*[laughed] (CS22)

### Purchasing from First Nations

A participant from Quebec who smoked 1–3 CPD talked about his experiences with purchasing cigarettes on Indian reservations: *“I don’t go online purchase. I usually go to the Indian’s reservations to buy cigarettes because they are cheaper there.”* He made these purchases with the knowledge that it was illegal for First Nations Canadians to sell cigarettes, *“The cigarettes that Indians make are very cheap because there’s no tax on them. But they are not licensed to sell. The law regulated their cigarette sales for only their own use.”* He was, however, not concerned about an illegal purchase because he had heard many Canadians who purchased cigarettes in this way were never bothered by the police.

### Roll-your-own cigarettes

A few participants resorted to purchasing loose tobacco to roll their own cigarettes to reduce the cost of smoking. One participant admitted to growing tobacco in his apartment, and vividly described the way he cared for the plant. According to him, rolling the tobacco leaves from his own plants had distinct benefits: *“It is obviously cheap to smoke this way. The tobacco leaves are very clean because these are the ones you have grown, right?”* He was also confident that his roll-your-own method was legal. *“You know the drugs, weed. Many people plant them at home too. That is illegal. But planting tobacco leaf is legal. The farmers in China usually plant tobacco for their own use. It’s like the same.”* Although he claimed that many other people around him did the same, none of the other Chinese participants mentioned having planted tobacco or even having heard of it. They were not sure about legality of this practice but suggested that people who had done the same might not tell outsiders if they perceived the practice as illegal.

## Discussion

This is the first study to explore how Chinese Canadian immigrant men who smoke access cigarettes. It provides valuable knowledge about the ways in which Chinese immigrants circumvent fully taxed cigarettes in Canada to continue their smoking.

This study supports the findings from previous research showing that although higher priced tobacco products may prompt low SES smokers to reduce or quit smoking, higher prices trigger the search for low or non-taxed options [8–10, 32]. The Chinese immigrant participants in this study resorted to some novel methods to satisfy their search for cheap cigarettes, a purchasing pattern that somehow deviates from the tobacco purchasing patterns of the mainstream population. A recent Canadian survey reported that smokers in Canada buy their cigarettes in the following ways: some 90% from a small grocery or convenience store or gas station, 6.5% from friend/family/someone else, 2.4% from First Nations reserves, and 2,2% from other sources [26]. While the participants, particularly the newer immigrants, might not be familiar with some of these purchasing patterns, they found unique means to buy cheap cigarettes by making use of their linkages to China. Strikingly, these Chinese immigrants were able to carry on purchasing cheap cigarettes on a long term basis. By accessing the cheap Chinese cigarettes, the Chinese immigrants were not only able to stick to their preferred cigarette tastes, but more importantly, afforded their smoking outside China.

Studies have found that people with high SES tend to buy overseas cigarettes simply because they travel overseas more often than those of lower SES [10]. Our study, however, identified how, despite their SES constraints, the Chinese immigrants also accessed cheap overseas cigarettes based on their international connections. The customs restrictions at Canadian borders did not influence the participants’ stock of Chinese cigarettes. Increased international travel among the Chinese participants’ extended family and friends facilitated their access to Chinese cigarettes across borders. The practice of accessing Chinese cigarettes has become an established behavior that involved the immigrants and their wider social networks. In this way, global travel patterns have outstripped the capacity for national and local regulatory frameworks to restrict tobacco. It is possible that within the context of international travel and international immigration patterns, tobacco purchasing patterns have reverted to patterns not unlike marijuana and other black market substances.

The influx of Chinese cigarettes brought across borders by Chinese immigrants and visitors has led to the online sales of Chinese cigarettes. There is a good possibility that the online market will grow with the inflow of more Chinese immigrants and visitors. It may develop beyond Chinese communities and attract mainstream smokers in Canada due to the extremely low price of Chinese cigarettes in this unregulated market. The participants expressed concerns about fake cigarettes with the surge of the online market and their concerns are justified. Produced in illegal cigarette factories in China, fake cigarettes in the form of “knock-off” Marlboros and other popular brands, are sold around the world. Lab tests indicate that these fake cigarettes contain higher levels of nicotine and toxic chemicals than brand-name cigarettes as well as contaminants [33].

Still, this demand for low-priced cigarettes will continue to fuel the presence of Chinese manufactured fake cigarettes available for online purchase. Other researchers have argued that the purchase of cheap online cigarettes is more likely to be used by people of higher SES, because it requires specific equipment and skills [9, 34]. However, as computers, including mobiles and wi-fi internet access is quickly becoming a norm of daily life for people from all backgrounds, online transactions in cigarettes will likely continue to grow.

Our study reveals that in addition to price-reduction, duty-free shop purchases reinforced the Chinese culture of gifting tobacco, which further enhanced the shopping motivations among the Chinese immigrants and their visitors. Other means of price-reduction purchases, such as purchasing cigarettes from neighboring USA, and purchasing cigarettes on First Nations reserves, were less likely to be used by participants in this study. This might be due to Chinese immigrants’ unfamiliarity with these sources or perhaps that their current sources had already met their needs. Still, there is a possibility that Chinese immigrants may explore other means of access the longer they live in Canada or as their smoking patterns change.

It should be pointed out that the flowing direction of cigarettes across borders may change with the change of exchange rates of currencies, which seem to have become more fluctuated in the past years. During the time the study was conducted in 2013, the value of Canadian dollar was also the same of USA dollar. The Canadian dollar has ever sense devalued compared to US dollar. The current US-Canada exchange rate is very unfavorable to Canadians and may mean that “border crossing” for cigarettes from USA to Canada is no longer attractive.

### Limitations

As with all qualitative research, caution is needed in generalizing the findings from this study to Chinese immigrants who smoke, not only because of the small number of the participants, but also because of the participants being a group of expectant fathers or fathers of young children. Also, the reliance on telephone interviews might have resulted in participants omitting information or communicating in ways that did not reflect the actual situation. For example, some participants might have perceived unconventional access to cigarettes as illegal and may have concealed their actual experiences.

### Implications for policies and research

As international efforts have attempted to curb illegal trade in tobacco, our study sheds light on how individuals legally obtain cheap cigarettes across borders and therefore sustain smoking prevalence among the immigrants. The current measures have not done enough to control the cross-border flow of cigarettes when each and every person travelling can legally carry cigarettes. A complete ban on tobacco crossing borders may be necessary.

Online purchase of cigarettes is a rapidly developing market in almost every country, and some nations have imposed restrictions. For example, in the USA, governments have collaborated with credit card companies to control online tobacco business [35]. The findings of the current study, however, pose new challenges to online sales because Chinese immigrants’ online purchases also involve off-line personal contacts. Monitoring and restrictions may need to be applied to website advertisements of tobacco products, regardless of language.

There are suggestions that tax increases should equally apply to factory-made and roll-your-own cigarettes [32]. While the First Nations people continue to offer tax-free sales of tobacco in their communities, there have been calls to implement taxation of tobacco sold to non-status Indians [8]. Increasing tobacco taxes in neighboring countries with lower cigarette prices has also been suggested. The European Union has urged to reduce price differences between the union countries, and to reduce the number of cigarettes or amount of loose leaf tobacco that a person can legally import for personal consumption [10]. However, the findings from our study suggest that imposing restrictions on the number of cigarettes that individuals can carry across borders is not likely to be sufficient to slow or stop the global movement of cigarettes. A complete ban on tobacco products across borders is necessary. However, it is not clear how countries could co-ordinate this effort and the extent to which it could stimulate the black market is unknown.

## Conclusions

The current study findings support the notion that price-reduced sources of cigarettes are popular among Chinese immigrants. It also indicates that the current cross-border regulations on cigarettes are being abused. Findings from this study support the WHO’s (2016) calls for policy makers, researchers, and the public to work together to curb the illicit trade of tobacco products. Our study is the first to provide in-depth knowledge about Chinese Canadian immigrant men’s access to cheap cigarettes. The findings of the study may have implications to other immigrants, because other immigrants may also seek low price cigarettes due to their similar social economic condition to Chinese immigrants. Future research studies could explore more detailed features of access to expose gaps in policy and improve tobacco regulatory frameworks.

## Abbreviations

CAD: Canadian dollar
CPD: Cigarettes per day
CS: Chinese smoker
RMB: Renminbi, Chinese dollars
SES: Social economic status
WHO: World Health Organization
